# Placebo and Nocebo Effects in Motor Performance: An Overview of Reviews

**DOI:** 10.1002/brb3.70534

**Published:** 2025-06-04

**Authors:** Cayque Brietzke, Wesley Alves Ribeiro, Paulo Estevão Franco‐Alvarenga, Raul Canestri, Ìtalo Vínicius, Gustavo Vasconcelos, Julio Cesario, Nelson Carvas Junior, Vitor de Salles Painelli, Pires Flávio Oliveira

**Affiliations:** ^1^ Exercise Psychophysiology Research Group. School of Arts Sciences and Humanities–University of São Paulo São Paulo Brazil; ^2^ Human Movement Science and Rehabilitation Program Federal University of São Paulo Santos Brazil; ^3^ Rehabilitation Sciences Program, Faculty of Medicine University of São Paulo São Paulo Brazil; ^4^ Physical Education Estácio de Sá University UNESA Resende Brazil; ^5^ Paulista University

**Keywords:** adverse effects, meta‐analysis, nocebo, placebo, psychomotor performance, research synthesis

## Abstract

**Objectives:**

To assess and synthesize the effect size and quality of the literature on the placebo and nocebo effects on motor performance and motor‐related perceptive responses.

**Design:**

Umbrella review.

**Data sources:**

Medline, Embase, Lilacs, Cochrane Database of Systematic Reviews for peer‐reviewed literature, PROSPERO for protocols, and the Open Access Theses and Dissertations for gray literature.

**Eligibility criteria for selecting studies:**

Population—human participants with varied health conditions; intervention: placebo; control: no treatment or active intervention; outcome: motor performance (primary) and perceptual variables (secondary); study design—ystematic reviews with or without meta‐analysis.

**Results:**

In total, 3432 records were gathered from searches, resulting in 13 eligible reviews after screening. These reviews encompassed 247 original studies, with 221 focusing on the placebo effect and 26 on the nocebo effect. Among all eligible systematic reviews, five conducted meta‐analysis with 5036 participants, and one provided a summary of effect sizes reported by the original studies with 1215 participants. The reviews reported small to large effects of placebo (SMD = 0.09–0.93) and nocebo (SMD = 0.37–1.20), and only two conducted the GRADE assessment.

**Conclusion:**

We found varied placebo and nocebo effects on motor performance, likely due to the poor quality of the methodology used by most reviews, highlighting the need for well‐conducted systematic reviews on the placebo and nocebo phenomena.

## Graphical abstract

This umbrella review assesses the methodological quality and certainty of evidence in systematic reviews on placebo and nocebo effects in motor performance. The findings reveal significant variability in effect sizes, ranging from small to large, and highlight concerns regarding the quality and bias in the existing literature.

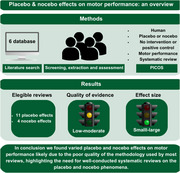

## 1 Introduction

The placebo effect is a response that arises when an individual believes that he/she is receiving a beneficial treatment, even though the treatment is inert (Finniss et al. 2010). This response arises from the expectation of receiving a beneficial treatment or from a conditioned response to a given stimulus (referred to as Pavlovian conditioning) (Brietzke et al. 2022). The magnitude of this response is associated with personality traits, social learning, and contextual factors (defined as perceived cues that directly influence the patient/volunteer and/or the clinician/researcher regarding the treatment received) (Finniss et al. 2010; Colloca and Benedetti 2006; Cook et al. 2023). Placebo effects may be observed in clinical and non‐clinical settings, as studies have shown that treatment‐derived expectation (or conditioning) can enhance motor performance, such as strength and endurance, as well as perceptual and electrophysiological outcomes, including pain, fatigue, sensation and brain activity (Finniss et al. 2010; Hurst et al. 2019; Zunhammer et al. 2018; Pires et al. 2018; C. Beedie et al. 2018; Rossettini et al. 2018). Although the underlying mechanisms are not completely understood, it has been suggested that placebo effects occur through a discharge in neurotransmitters such as dopamine, glutamate, and endogenous opioids that modify the activation of neuronal tissue involved in cerebral reward and motor planning and command systems (Brietzke et al. 2022; De la Fuente‐Fernández et al. 2001). Therefore, rather than being inert, effects induced by treatment expectations or conditioning may alter psycho‐neurophysiological variables that potentially affect motor system behavior.

Original studies have reported varied effect sizes of placebo effects in outcomes of motor domains, thus producing controversial conclusions on the magnitude of the placebo effects on motor performance (Hurst et al. 2019; Palma et al. 2022; Gallone et al. 2022; Chhabra and Szabo 2024; Rawdon 2006). For example, we observed large improvements (11.9%–17.4%) in maximal exercise performance when individuals were induced to believe they were ingesting a powerful ergogenic aid such as caffeine (Pires et al. 2018; Brietzke et al. 2017). However, we observed lower improvements in motor performance (3.8%–6.6%) in placebo effects derived from the individual's appraisal of the benefit of ingesting a beverage with higher energy content (de Salles Painelli et al. 2023). Varying effect sizes have been reported by other studies investigating placebo effects in different motor performance scenarios (Pires et al. 2018; Rossettini et al. 2018; de Salles Painelli et al. 2023). In this regard, systematic reviews and meta‐analysis studies (SRMAs) assessing placebo effects in various populations, including patients with chronic coronary syndrome (Palma et al. 2022) and healthy subjects (Marticorena et al. 2021), have found varied effect sizes in motor performance outcomes and motor system‐related perceptual variables such as pain and fatigue sensation, thereby creating uncertainty about the magnitude of the placebo effects.

Discrepancies observed in the placebo effects assessed by different SRMA studies may also be attributed to the diversity of methodological approaches used for review. For example, SRMAs have used several different criteria for selecting original studies (Hurst et al. 2019; Palma et al. 2022; Gallone et al. 2022; Rawdon 2006; Marticorena et al. 2021) and estimating the pooled effect size of placebo effects (Hurst et al. 2019; Palma et al. 2022; Gallone et al. 2022; Marticorena et al. 2021; Motoyama 2019). Consequently, scientists and professionals in clinical fields face difficulties in summarizing the true placebo effects on motor system outcomes when consulting different SRMA studies. Importantly, umbrella reviews are useful for aggregating divergent results reported by SRMAs, as well as for identifying methodological inconsistencies. This approach is feasible for grouping and summarizing the varied findings on placebo effects in motor performance and related perceptual responses, while also assessing the certainty of evidence and estimating the magnitude of placebo effects across a comprehensive sample.

Therefore, the present umbrella review assessed the methodological quality and certainty of the evidence of previous SRMAs on placebo effects in motor system outcomes. Our aim was to aggregate the varied effect sizes of placebo effects in primary outcomes such as motor performance, as well as secondary outcomes such as pain and fatigue sensation.

## 2 Methods

This review was conducted according to the recommendations of the Joanna Briggs Institute and Cochrane Handbook (Higgins et al. 2022; Aromataris and Munn 2020) and reported according to the Preferred Reporting Items for Systematic Reviews and Meta‐Analyses (PRISMA; https://prisma.shinyapps.io/checklist/) (Page et al. 2021), Perspectives for Reporting Systematic Reviews in Sport Science (PERSiST) (Ardern et al. 2022), and Preferred Reporting Items for Overviews of Reviews (PRIOR) (Gates et al. 2022) statements. This review was previously registered in PROSPERO: International Prospective Register of Systematic Reviews (number #42021270112). There were no major protocol violations.

### 2.1 Equity, Diversity, and Inclusion Statement

Our research team comprises individuals from diverse socio‐cultural backgrounds, including diversity in race, gender, sexual orientation, beliefs, geographic locations, socio‐economic levels, and career stages. We do not make distinctions based on these factors nor use them as criteria for establishing authorship.

### 2.2 Eligibility Criteria, Data Sources, and Searches

Systematic reviews, whether they included meta‐analysis or not, were considered eligible based on PICOS criteria as outlined in Table 1. All searches were conducted on a single day (September 23, 2022) and updated on May 04, 2024, without language restrictions. We searched for peer‐reviewed literature indexed in Medline via PubMed, Embase, Lilacs via Virtual Health Library (VHL), and the Cochrane Database of Systematic Reviews. Additionally, we searched the PROSPERO database for review protocols and the Open Access Theses and Dissertations for gray literature (Aromataris and Munn 2020). The references of the reviews included were also checked. The search strategy used in PubMed (Table 2) was adapted for the other databases, as detailed in Supporting Information S1.

**TABLE 1:** PICOS (participants, intervention, comparator, outcomes, study design) criteria.

PICO	Details
Participants	Human participants from varied health profiles
Intervention	Placebo
Comparator	Control (a group receiving no treatment) or positive control (a group receiving active treatment).
Outcomes	Primary outcomes: motor performance responses
	Secondary outcomes: pain and fatigue sensation and electrophysiological variables
Study design	Systematic reviews

**TABLE 2:** Search strategy in MEDLINE via Pubmed.

Search number	Combiners	Search terms
#1	Research interest	"Placebo Effect"[MeSH Terms] OR ("Placebo Effect"[MeSH Terms] OR ("placebo"[All Fields] AND "effect"[All Fields]) OR "Placebo Effect"[All Fields] OR ("effect"[All Fields] AND "placebo"[All Fields]) OR "effect placebo"[All Fields]) OR ("Placebo Effect"[MeSH Terms] OR ("placebo"[All Fields] AND "effect"[All Fields]) OR "Placebo Effect"[All Fields] OR ("placebo"[All Fields] AND "response"[All Fields]) OR "placebo response"[All Fields]) OR ("Placebo Effect"[MeSH Terms] OR ("placebo"[All Fields] AND "effect"[All Fields]) OR "Placebo Effect"[All Fields] OR ("response"[All Fields] AND "placebo"[All Fields]) OR "response placebo"[All Fields]) OR ("placebos"[MeSH Terms] OR "placebos"[All Fields] OR ("sham"[All Fields] AND "treatment"[All Fields]) OR "sham treatment"[All Fields]) OR "Nocebo Effect"[MeSH Terms] OR ("Nocebo Effect"[MeSH Terms] OR ("nocebo"[All Fields] AND "effect"[All Fields]) OR "Nocebo Effect"[All Fields] OR ("effect"[All Fields] AND "nocebo"[All Fields]) OR "effect nocebo"[All Fields]) OR ("Nocebo Effect"[MeSH Terms] OR ("nocebo"[All Fields] AND "effect"[All Fields]) OR "Nocebo Effect"[All Fields] OR ("effects"[All Fields] AND "nocebo"[All Fields]) OR "effects nocebo"[All Fields]) OR ("Nocebo Effect"[MeSH Terms] OR ("nocebo"[All Fields] AND "effect"[All Fields]) OR "Nocebo Effect"[All Fields] OR ("nocebo"[All Fields] AND "effects"[All Fields]) OR "nocebo effects"[All Fields]) OR ("Nocebo Effect"[MeSH Terms] OR ("nocebo"[All Fields] AND "effect"[All Fields]) OR "Nocebo Effect"[All Fields] OR "nocebo"[All Fields] OR "nocebos"[All Fields])
#2	Type of study	((((((((((((((((("systematic review"[Title] OR "systematic literature review"[Title]) OR "systematic scoping review"[Title]) OR "systematic narrative review"[Title]) OR "systematic qualitative review"[Title]) OR "systematic evidence review"[Title]) OR "systematic quantitative review"[Title]) OR "systematic meta review"[Title]) OR "systematic critical review"[Title]) OR "systematic mixed studies review"[Title]) OR "systematic mapping review"[Title]) OR "systematic cochrane review"[Title]) OR "systematic search and review"[Title]) OR "systematic integrative review"[Title]) NOT "comment"[Publication Type]) NOT ("protocol"[Title] OR "protocols"[Title])) NOT "MEDLINE"[Filter]) OR ("cochrane database syst rev"[Journal] AND "review"[Publication Type])) OR "systematic review"[Publication Type]
#3		(#1) AND (#2)

*Note*: Filter proposed by Hayne (Haynes RB, McKibbon KA, Wilczynski NL, Walter SD, Werre SR; Hedges Team. Optimal search strategies for retrieving scientifically strong studies of treatment from Medline: analytical survey. BMJ. 2005 May 21;330(7501):1179. doi: 10.1136/bmj.38446.498542.8F. Epub 2005 May 13. PMID: 15894554; PMCID: PMC558012.).

### 2.3 Primary and Secondary Outcomes

Measures of motor performance, such as time to exhaustion, power output, and so forth, were the primary outcome, while sensations of pain or fatigue were the secondary ones. We also included motor system‐related electrophysiological responses as a secondary outcome, as they may offer valuable information into the placebo effects on motor systems’ functioning. Indeed, assuming the likely underlying mechanisms of placebo and nocebo effects on motor performance (Brietzke et al. 2022), these secondary outcomes may improve the understanding of the placebo phenomenon. Given that their responses are specific to a given placebo intervention, they were included only if they were reported together as the primary outcomes.

### 2.4 Screening and Data Extraction

Two pairs of investigators (CB with PEFA and GV with RC) selected eligible reviews in two steps using the Rayyan application (Ouzzani et al. 2016). The investigators independently screened titles and abstracts (Step 1) before reviewing the full texts of eligible studies. To reduce the chances of disagreements between investigators, we conducted several pilot trials with a small sample of studies to standardize the procedures used to screen eligible studies and extract data. Eventually, disagreements were solved by a senior investigator (FOP). A pair of investigators (CB and RC) individually extracted data from eligible reviews using a standardized spreadsheet as suggested by the Joanna Briggs Institute Manual for Evidence Synthesis (Aromataris and Munn 2020). Another investigator (WAR) checked for inconsistencies in data extraction and searched for additional information when necessary.

### 2.5 Methodological Quality and Certainty of Evidence in Included Reviews

The methodological quality of the SRMAs was assessed by two investigators (CB and WAR) using the Assessing the Methodological Quality of Systematic Reviews 2 (AMSTAR 2) checklist (Shea et al. 2017). This checklist includes 16 items that evaluate whether the SRMA reported the review design prior to conducting the review, applied the PICOS criteria, and adequately performed data extraction and quantitative synthesis. We also evaluated the certainty of evidence for the primary outcomes included in this umbrella if previous SRMA made them available. Any disagreements were resolved by a senior investigator (FOP).

### 2.6 Data Synthesis

We used the effect size indexes as reported by the SRMA themselves, except for one study (Rooney et al. 2024) that separately analyzed physical and motor performance, prompting us to reanalyze them by pooling all relevant studies together. Additionally, we calculated the percentage of non‐null effects in primary studies (studies reporting significant effects of placebo or nocebo) before calculating the overlap of primary studies as early suggested (Pieper et al. 2014).

## 3 Results

**FIGURE 1:**
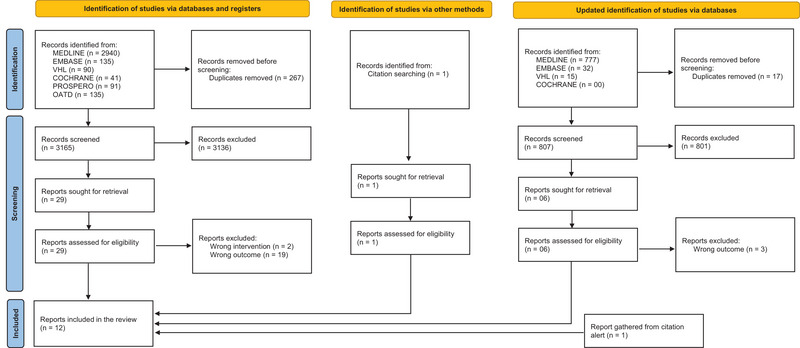
Flowchart of the selection procedures.

The characteristics of the studies are presented in Table 3. An initial comprehensive search yielded a total of 3432 studies, from which 13 eligible reviews were identified after screenings. The update retrieved 824 studies, of which 3 were eligible, and an additional study was included via citation notification on ResearchGate. These reviews assessed 302 original studies, comprising 245 investigations of placebo effects and 42 investigations of nocebo effects. In total, these reviews included 12,145 participants, with 9790 in placebo interventions and 3111 in nocebo interventions. Among all reviews, 7 were SRMAs that meta‐analyzed 5,762 participants. Additionally, one review provided a summary of effect sizes reported by original studies, including 950 participants in placebo and 265 participants in nocebo (Table 3). Interestingly, we observed a variety of outcomes reported as “motor performance.” While some reported motor performance as “exercise time” assessed in protocols frequently used in clinical scenarios such as Bruce's and modified Bruce's VO_2MAX_ test, others reported measures such as speed, power output, time of exercise, or maximal weight during different exercise protocols (Hurst et al. 2019; Chhabra and Szabo 2024; Rawdon 2006; Marticorena et al. 2021; Motoyama 2019; Bérdi et al. 2011; Spille et al. 2023). Measures such as speed and power output represented ∼62% of the reported outcomes (8/13 studies), while motor performance assessed as exercise time represented ∼15% of the reported outcomes (2/13). Importantly, none of these reviews reported perceptual or electrophysiological variables related to motor performance; thus we could not report pain and fatigue sensation as a secondary outcome in this umbrella review. The flowchart summarizing details of the SRMA selection procedure is presented in Figure 1.

**TABLE 3:** Summary characteristic of reviews

Review	Population	Intervention	Comparator	Design of included studies^*^	Number of included studies (whole sample size)	Effect size (95%CI)	I^2^	Review‐reported GRADE
Palma et al. (2022)	Patients class I‐IV angina	Placebo	Invasive treatments for chronic coronary syndrome	RCT	13 studies (n = 454)	0.22 (0.09;0.35)	0%	Low
Sheriff et al. (2022)^a^	Patients with chronic low back pain	Placebo	Conservative strategies wich can improve outcome due contextual factor	RCT and NRSI	21 studies (n = 3075)	NA	NA	NR
Gallone et al. (2022)	Patients with symptomatic stable angina CAD	Placebo	CAD conventional treatment	RCT	62 studies (n = 3891)	29.20 (20.60;37.80)	98%	NR
Marticorena et al. (2021)	Healthy humans	Placebo	Caffeine and buffer suplement	RCT	34 studies (n = 363)	0.09 (0.01; 0.17)	NA	NR
Hurst et al. (2019)^a^	Participants described as "apparently healthy" or "athletes"	Placebo	Ergogenic aids were categorised into nutritional and mechanical	RCT	32 studies (n = 950)	0.36 (0.21;0.50)	NR	NR
Hurst et al. (2019)^a^	Participants described as "apparently healthy" or "athletes"	Nocebo	Ergogenic aids were categorised into nutritional and mechanical	RCT	5 studies (n = 265)	0.37 (0.12;0.61)	NR	NR
Motoyama et al. (2019)^[Link]^, ^b^	Healthy humans	Placebo	Physical performance ergogenic treatment	RCT	8 studies (n = 91)	NA	NA	Very low
Horváth et al. (2021)	Any human population, healthy and unhealthy	Nocebo	Noneffective sham transcutaneous stimulation and inert substances	RCT	21 studies (n = 1953)	NA	NA	NR
Rawdon et al. (2006)^b^	Healthy humans	Placebo	Nutrcional supplemetation	RCT	37 studies (n = 132)	0.67 (‐0.09;1.44)	NR	NR
Bérdi et al. (2011)	Healthy humans	Placebo	Ergogenic intervention in sporting performance	RCT	14 studies (n = 196)	0.31 (0.24;0.56)	NR	NR
Spille et al. (2023)^a^	Healthy humans	Open‐label placebo	No‐treatnebt control condition or covert placebo	RCT	2 studies (n = 49)	NR	NA	NR
Żegleń et al. (2024)	Pediatric population	Placebo	The intervention used in the control group (e.g., no‐treatment or natural history group)	RCT	3 studies (n = 68)	0.93 (0.57;1.28)	0%	NR
Rooney et al. (2024)	Healthy and unhealthy population	Nocebo	Inactive agent or no‐treatment	RCT	10 studies (n = 658)	0.54 (0.32;0.77)	46.5%	NR
Chhabra & Szabo (2024)^a^	Recreational exercisers, athletes and healthy individuals	Placebo	Nutritional, mechanical, or other characteristics, including verbal manipulation.	RCT	19 studies (n = 521)	NR	NR	NR
Chhabra & Szabo (2024)^a^	Recreational exercisers, athletes and healthy individuals	Nocebo	Nutritional, mechanical, or other characteristics, including verbal manipulation.	RCT	6 studies (n = 235)	NR	NR	NR

^a^ no meta‐Analysis.

^b^ gray literature; NA = not applicable

* as defined by AMSTAR 2; RCTs = randomized controlled trials; NRSI = non‐randomized studies of interventions; NR = not reported; CAD = coronary artery disease

### 3.1 Methodological Quality and Certainty of Evidence

**FIGURE 2:**
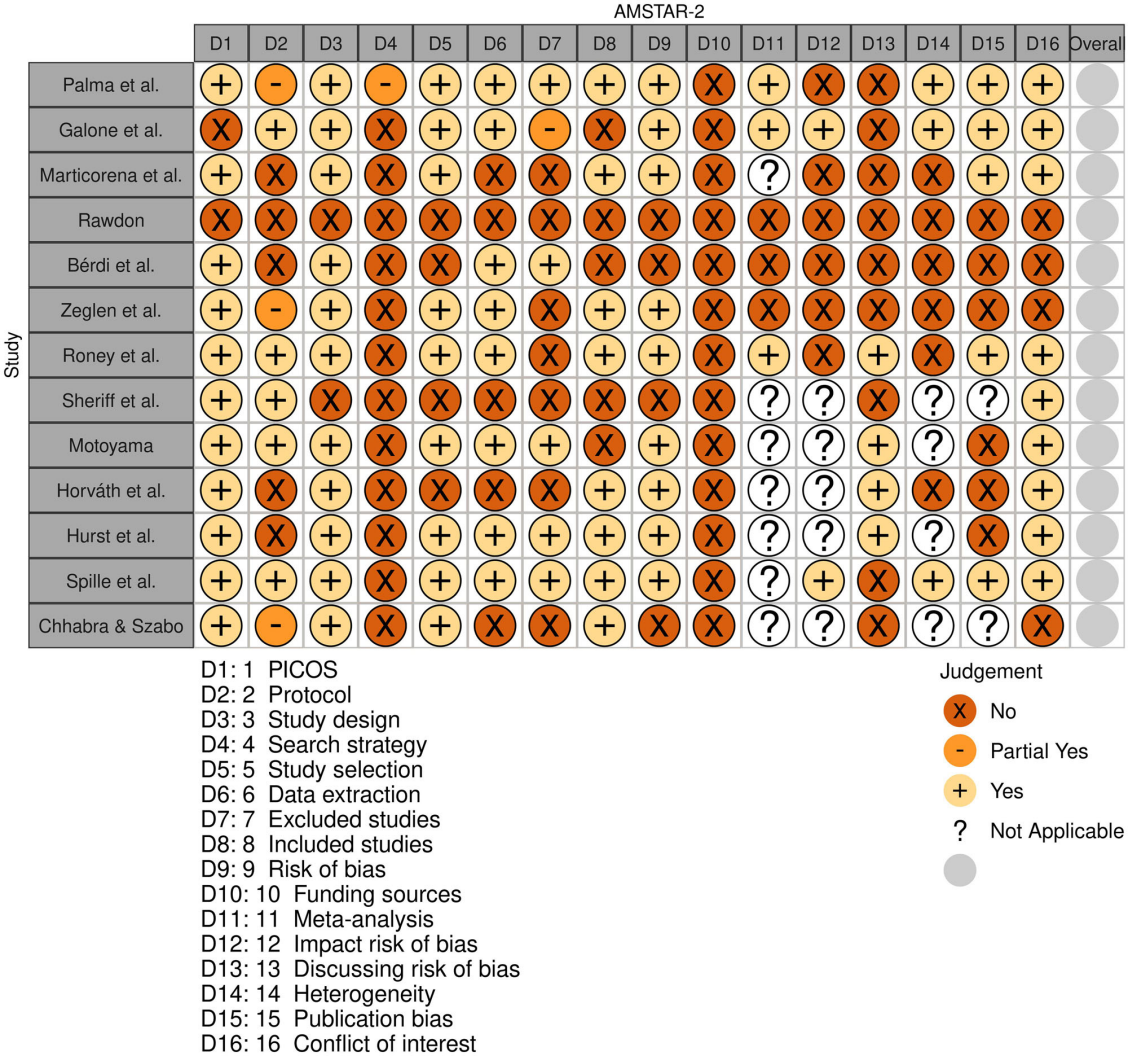
Methodological quality of the literatures assessed through the AMSTAR 2.

The methodological quality of the SRMAs included in this umbrella varied from low to moderate, as none of them adequately addressed all critical items designed to evaluate SRMAs. Frequently, Items 1 and 16 were unaddressed by the reviews. Moreover, items evaluating protocol registration and deviations (Item 2), comprehensive literature search (Item 4), and reporting and discussion of the risk of bias (RoB) in primary studies (Items 12 and 13) were inadequately addressed by most reviews, representing significant concerns. The methodological quality of the reviews is presented in Figure 2. Importantly, the Grading of Recommendations Assessment, Development, and Evaluation (GRADE) analysis conducted by Palma et al. (2022) and Motoyama (2019) identified significant limitations regarding RoB and inconsistencies, such as the elevated heterogeneity and no overlap across confidence intervals in original placebo and nocebo studies.

### 3.2 Placebo and Nocebo Effects

Out of the 13 reviews, only 7 conducted meta‐analysis (Palma et al. 2022; Gallone et al. 2022; Rawdon 2006; Marticorena et al. 2021; Rooney et al. 2024; Bérdi et al. 2011; Żegleń et al. 2024), 3 reviews reported estimated effect sizes (Hurst et al. 2019; Chhabra and Szabo 2024; Horváth et al. 2021), a single review reported individual effect sizes from original studies (Aromataris and Munn 2020), while the other review did not report effect sizes (Sherriff et al. 2022). One review did not include studies about physical performance in meta‐analysis because these studies used a “within‐subject” design (Spille et al. 2023). Among reviews with meta‐analysis, one presented the effect size as a mean difference in performance (29.2 s; 95% CI [20.6–37.8]) measured in a treadmill exercise test (Gallone et al. 2022), while six reported the effect as a standardized mean difference with pooled effects ranging from 0.09 (Marticorena et al. 2021) to 0.93 (Żegleń et al. 2024). The only study to find null effects of placebo in performance measurements was by Rawdon (2006). Regarding heterogeneity, only studies by Palma et al. (2022) and Żegleń et al. (2024) reported *I* ^2^ = 0%, while Gallone et al. (2022) and Rooney et al. (2024) reported *I* ^2^ = 98% and 46.5 %, respectively. A review by Marticorena et al. (2021) conducted a Bayesian analysis that incorporated the heterogeneity.

The reviews by Hurst et al. (2019) and Horváth et al. (2021) reported estimated effect sizes rather than meta‐analysis. Hurst et al. (2019) estimated the placebo effect size as *d* = 0.36 ± 0.44, 95% CI (0.21–0.50), and the nocebo effect size as *d* = 0.37 ± 0.25, 95% CI (0.12–0.61). Horváth et al. (2021) estimated the nocebo effect size as *d* = 0.60 without providing interval estimation precision. The study by Motoyama (2019) presented placebo effect sizes as reported by individual original studies (−0.5–1.7).

Analysis revealed varied proportions of non‐null placebo (Hurst et al. 2019; Chhabra and Szabo 2024; Motoyama 2019; Sherriff et al. 2022) and nocebo effects among studies (Hurst et al. 2019; Chhabra and Szabo 2024). For example, Hurst et al. (2019) reported the highest positive proportion of placebo effects (76.7%), as 23 out of 30 studies revealed beneficial effects of placebo. In contrast, a review by Motoyama (2019) found the lowest positive proportion of placebo effects (12.5%) in 1 out of 8 studies. Chhabra and Szabo (2024) recorded 57.9% of positive effects, whereas Spille et al. (2023) and Sherriff et al. (2022) presented equal proportions (50%). Regarding nocebo effects, Hurst et al. (2019) showed the highest positive proportion of harmful effects of nocebo (80%), with four out of five studies reporting nocebo effects. Accordingly, a review by Chhabra and Szabo (2024) (66.7%) found the harmful effect of a placebo in 4 out of 6 reviews. Analysis of overlap over primary studies revealed a slight overlap (0.02) of studies included by reviews.

## 4 Discussion

In the present study, we conducted an overview of placebo and nocebo effects on motor performance outcomes. We identified varying levels of quality in SRMA studies, ranging from low to moderate. Furthermore, based on only two reviews, we found that the certainty of evidence regarding placebo and nocebo effects on motor performance was low to very low. These results are discussed below, highlighting the need for robust SRMAs investigating the placebo and nocebo effects on motor performance.

### 4.1 Quality and Certainty of Evidence in Placebo and Nocebo Literature

We identified concerns in the placebo and nocebo literature, as only a few reviews met the quality necessary to address at least half of the checklist items (Hurst et al. 2019; Palma et al. 2022; Gallone et al. 2022; Motoyama 2019). Most importantly, we found that a substantial number of SRMAs exhibited important bias. For instance, assessments of protocol registration (Item 2), study design (Item 3), comprehensiveness of the literature search (Item 4), and RoB assessment (Items 12 and 13) revealed that reviews on placebo effects on motor performance have been poorly conducted. Although AMSTAR‐2 is not intended to provide an overall score for reviews in the field (Shea et al. 2017), the results of this checklist highlighted the necessity of well‐designed reviews in the placebo literature.

Of the 13 included reviews, only 5 fully adhered to the pre‐published protocol criterion (Gallone et al. 2022; Motoyama 2019, Rooney et al. 2024; Spille et al. 2023; Sherriff et al. 2022). Prior registration of methods in a systematic review is particularly important to ensure transparency throughout the review process. For instance, assessing deviations from the intended intervention and analysis is hindered when the methods are not declared beforehand in a registered protocol, potentially rendering the results reported by the review unreliable. Unfortunately, in five reviews this information was lacking (Hurst et al. 2019; Rawdon 2006; Marticorena et al. 2021; Bérdi et al. 2011; Horváth et al. 2021), while three reviews partially met this criterion (Palma et al. 2022; Chhabra and Szabo 2024; Żegleń et al. 2024). For instance, none of the reviews fully met the criteria for a comprehensive search for literature. Despite the assessment of this item being quite rigorous (requiring the fulfillment of eight items to be considered “good”), the main issue with the comprehensiveness of the search literature was the restrictions placed on publications searching (such as language) without any justification. The minimal requirement in this regard is particularly important to prevent the possibility of “cherry‐picking” and to encompass the entire literature by utilizing multiple databases, gray literature, and a broad scope of publications in various languages. In the placebo literature assessed in our review, the most common weaknesses regarding the comprehensiveness of the search were unjustified restrictions, absence of expert consultation, and absence of searching in registry databases.

### 4.2 Placebo and Nocebo Effect on Motor Performance. A Quantitative Analysis

As far back as 1970, research by Ariel and Saville (1972) demonstrated that individuals who believed they were receiving anabolic steroids during a strength training program experienced greater strength gains compared to those who trained without them. The current umbrella review confirmed that expecting a positive effect from a treatment may significantly enhance motor performance, as demonstrated by original studies exploring different interventions (Pires et al. 2018; C. J. Beedie et al. 2008; Foad et al. 2008; Pollo et al. 2008) and assessing various motor performance outcomes in a variety of scenarios (Pires et al. 2018; Pollo et al. 2008; Fiorio et al. 2014). While not fully understood, the mechanisms underlying the placebo effects may involve various psycho‐neurochemical routes. For instance, we recently suggested that the placebo effect may be triggered by psychological events, such as various forms of declarative expectancy (Brietzke et al. 2022). This would initiate the release of different neurotransmitters, including dopamine, glutamate, and endogenous opioids within reward circuit areas connected to motor‐related cortical areas such as the prefrontal cortex, supplementary motor areas, and motor cortex (Brietzke et al. 2022).

We reviewed SRMAs involving more than 9000 participants. The findings confirmed that placebo effects may enhance motor performance across a range of health conditions, such as in cardiac patients and athletes. However, the literature from this field is highly heterogeneous, with effect sizes ranging from small to large (SMD = 0.09−0.93). These differences can be attributed to factors such as the type of intervention (e.g., supplement type, mechanical or nutritional intervention, etc.) (Hurst et al. 2019; Marticorena et al. 2021), as well as the form of its administration (Marticorena et al. 2021). Interestingly, a recent review revealed that studies conducted before the 2000s reported stronger placebo effects than studies published more recently (Marticorena et al. 2021). Perhaps this disparity stems from improved blinding strategies used in more recent studies, as the awareness of the treatment received can influence individuals’ behavior, potentially inducing bias in outcomes typically measured in exercise studies (Saunders et al. 2016; Painelli V de et al. 2020). Therefore, implementing effective procedures for blind participants and research personnel involved in the trial is crucial for controlling this source of bias and providing reliable results.

Unlike placebos, nocebo's effects on motor performance have been largely overlooked. Recently, a task force has drawn attention to the necessity of investigating the effects of negative expectations on physical performance (C. Beedie et al. 2018). Indeed, we found a scarcity of studies on nocebo effects, as we only identified three reviews exploring these effects (Hurst et al. 2019; Chhabra and Szabo 2024, Horváth et al. 2021). Furthermore, neither of these reviews conducted an adequate meta‐analysis, which is crucial for accurately estimating the weighted effect sizes, assessing study heterogeneity, and evaluating other factors such as potential publication bias and the effect size by RoB tools. These three systematic reviews assessed the nocebo effects of more than 2000 participants (Hurst et al. 2019; Chhabra and Szabo 2024; Horváth et al. 2021); however, they estimated the nocebo effect sizes without performing a meta‐analysis. For instance, Hurst et al. (2019) reported a small effect size (*d* = 0.37) when participants held negative beliefs, while the more recent reviews by Horváth et al. (2021) and Chhabra and Szabo (2024) reported effect size estimates of 0.60 and 1.20, respectively, indicating a stronger nocebo effect compared to the earlier study. However, neither of these reviews provided insights into the heterogeneity of the literature or the consistency of these effects across potential subcategories, as commonly examined in placebo meta‐analyses.

Another two important points should be highlighted. Firstly, we included motor system‐related perceptual and electrophysiological responses as secondary outcomes, as they may offer valuable information into the placebo effects on motor systems’ functioning (Brietzke et al. 2022). However, none of the retrieved reviews reported these variables alongside motor performance responses, thus limiting a better understanding of how perceptual and electrophysiological responses are associated with placebo effects in motor performance. Future SMRA may consider including pain, fatigue, and neurophysiological variables to help a better understanding of the relationship between motor performance and psycho‐neurophysiological responses in placebo scenarios. Secondly, placebo and nocebo effects on motor performance may be highly dependent on rituals of clinical or experimental procedures, including verbal instructions, characteristics of the vehicle used as an intervention, exercise testing, and motor performance assessment (Pires et al. 2018; Brietzke et al. 2017; Fiorio et al. 2014; Fiorio 2018). Hence, future SRMA should analyze these aspects to improve the precision when estimating placebo and nocebo effects on motor performance. In this regard, clinical professionals and researchers should be able to precisely quantify the effects of different forms of inducing placebo on performance outcomes assessed through distinct instruments. So far, they cannot precisely quantify placebo and nocebo influences on motor performance.

### 4.3 Limitations

Two potential limitations of this overview should be pointed out. Firstly, we calculated neither the meta‐analysis ‐derived weighted effect sizes nor effect size indexes for SRMA that eventually did not report these results. However, we calculated the proportion of original studies reporting beneficial placebo effects in relation to the total number of studies retrieved by those reviews, somehow covering this gap. Furthermore, as GRADE assessments were reported by only two retrieved reviews, the certainty of evidence in placebo literature may be considered rather inaccurate.

In summary, the present overview found small to large placebo and nocebo effects on motor performance. However, the quality of the included reviews should be questioned, as they did not adequately fulfill important quality criteria and did not assess the certainty of the available evidence. Future systematic reviews on placebo and nocebo effects should use quality assessment tools for SRMA.

## Author Contributions

**Cayque Brietzke**: conceptualization, investigation, writing – original draft, methodology, formal analysis. **Wesley Alves Ribeiro**: investigation, methodology, formal analysis, data curation, writing – review and editing. **Paulo Estevão Franco‐Alvarenga**: investigation, methodology, formal analysis, data curation, writing – review and editing. **Raul Canestri**: investigation, methodology, formal analysis, data curation, writing – review and editing. **Ìtalo Vínicius**: investigation, methodology, formal analysis, data curation, writing – review and editing. **Gustavo Vasconcelos**: investigation, methodology, formal analysis, data curation, writing – review and editing. **Julio Cesario**: investigation, methodology, writing – review and editing, formal analysis, data curation. **Nelson Carvas Junior**: validation, formal analysis, writing – review and editing, conceptualization. **Vitor de Salles Painelli**: investigation, methodology, writing – review and editing, formal analysis, data curation. **Pires Flávio Oliveira**: conceptualization, funding acquisition, writing – original draft, writing – review and editing, project administration, supervision, resources.

## Ethics Approval

The authors have nothing to report.

## Consent

The authors have nothing to report.

## Conflicts of Interest

The authors declare no conflicts of interest.

## Peer Review

The peer review history for this article is available at https://publons.com/publon/10.1002/brb3.70534

## Supporting information



Supporting Information

## Data Availability

Data is available upon reasonable request.
