# Supplementary material for: Placebo and Nocebo Effects in Motor Performance: An Overview of Reviews
Source: Brain Behav. 2025 Jun 4;15(6):e70534. doi: 10.1002/brb3.70534 (PMC12134487; doi:10.1002/brb3.70534)
Supplement: Supplementary file 1 — Supporting Information [file BRB3-15-e70534-s001.pdf]

"Placebo Effect"[MeSH Terms]

"Nocebo Effect"[MeSH Terms]

"Placebos"[Mesh]

"Motor Activity"[Mesh]

"Exercise"[Mesh]

"Pain"[Mesh]

"Pain Perception"[Mesh]

"Fatigue"[Mesh]

"Electrophysiology"[Mesh]

"Electrophysiological Phenomena"[Mesh]

==///==============
==================

PUBMED

==///==============
==================

#1 -

((((((((((((((("systematic review"[Title] OR "systematic literature review"[Title]) OR "systematic scoping review"[Title]) OR "systematic narrative review"[Title]) OR "systematic qualitative review"[Title]) OR "systematic evidence review"[Title]) OR "systematic quantitative review"[Title]) OR "systematic meta review"[Title]) OR "systematic critical review"[Title]) OR "systematic mixed studies review"[Title]) OR "systematic mapping review"[Title]) OR "systematic cochrane review"[Title]) OR "systematic search and review"[Title]) OR "systematic integrative review"[Title]) NOT "comment"[Publication Type]) NOT ("protocol"[Title] OR "protocols"[Title])) NOT "MEDLINE"[Filter]) OR ("cochrane database syst rev"[Journal] AND "review"[Publication Type])) OR "systematic review"[Publication Type]

#2

"Placebo Effect"[Mesh] OR (Effect, Placebo) OR (Placebo Response) OR (Response, Placebo) OR "Nocebo Effect"[Mesh] OR (Effect, Nocebo) OR (Effects, Nocebo) OR (Nocebo Effects) OR (Nocebo) OR "Placebos"[Mesh] OR (Sham Treatment)

#3

"Motor Activity"[Mesh] OR (Activities, Motor) OR (Activity, Motor) OR (Motor Activities) OR "Exercise"[Mesh] OR (Exercises) OR (Physical Activity) OR (Activities, Physical) OR (Activity, Physical) OR (Physical Activities) OR (Exercise, Physical) OR

(Exercises, Physical) OR (Physical Exercise) OR (Physical Exercises) OR (Acute Exercise) OR (Acute Exercises) OR (Exercise, Acute) OR (Exercises, Acute) OR (Exercise, Isometric) OR (Exercises, Isometric) OR (Isometric Exercises) OR (Isometric Exercise) OR (Exercise, Aerobic) OR (Aerobic Exercise) OR (Aerobic Exercises) OR (Exercises, Aerobic) OR (Exercise Training) OR (Exercise Trainings) OR (Training, Exercise) OR (Trainings, Exercise)

#4

"Pain"[Mesh] OR (Pain, Burning) OR (Burning Pain) OR (Burning Pains) OR (Pains, Burning) OR (Suffering, Physical) OR (Physical Suffering) OR (Physical Sufferings) OR (Sufferings, Physical) OR (Pain, Migratory) OR (Migratory Pain) OR (Migratory Pains) OR (Pains, Migratory) OR (Pain, Radiating) OR (Pains, Radiating) OR (Radiating Pain) OR (Radiating Pains) OR (Pain, Splitting) OR (Pains, Splitting) OR (Splitting Pain) OR (Splitting Pains) OR (Ache) OR (Aches) OR (Pain, Crushing) OR (Crushing Pain) OR (Crushing Pains) OR (Pains, Crushing) OR"Pain Clinics"[Mesh] OR (Clinic, Pain) OR (Clinics, Pain) OR (Pain Clinic) OR (Pain Relief Units) OR (Pain Relief Unit) OR(Unit, Pain Relief) OR (Units, Pain Relief) OR (Pain Centers) OR (Center, Pain) OR (Centers, Pain) OR (Pain Center) OR(Multidisciplinary Pain Centers) OR (Center, Multidisciplinary Pain) OR (Centers, Multidisciplinary Pain) OR (Multidisciplinary Pain Center) OR (Pain Center, Multidisciplinary) OR (Pain Centers, Multidisciplinary) OR (Multidisciplinary Pain Clinics) OR(Clinic, Multidisciplinary Pain) OR (Clinics, Multidisciplinary Pain) OR (Multidisciplinary Pain Clinic) OR (Pain Clinic, Multidisciplinary) OR (Pain Clinics, Multidisciplinary) OR (Acute Pain Service) OR (Pain Service, Acute) OR (Acute Pain Services) OR (Pain Services, Acute) OR (Service, Acute Pain) OR (Services, Acute Pain) OR "Pain Perception"[Mesh] OR (Pain Perceptions) OR (Perception, Pain) OR (Perceptions, Pain)

#5

"Fatigue"[Mesh] OR (Lassitude)

#6

"Electrophysiology"[Mesh] OR "Electrophysiological Phenomena"[Mesh] (Phenomena, Electrophysiological) OR (Electrophysiologic Concepts) OR (Concept, Electrophysiologic) OR (Concepts, Electrophysiologic) OR (Electrophysiologic Concept) OR (Electrophysiologic Phenomena) OR (Phenomena, Electrophysiologic) OR (Electrophysiological Phenomenon) OR (Phenomenon, Electrophysiological) OR (Electrophysiological Concepts) OR (Concept, Electrophysiological) OR (Concepts, Electrophysiological) OR (Electrophysiological Concept) OR (Electrophysiological Processes) OR (Processes, Electrophysiological) OR (Electrophysiological Process) OR (Process, Electrophysiological)

#7

(#1) AND (#2) AND (#3 OR #4 OR #5 OR #6)

===///============================

EMBASE

===///============================

'nocebo effect'/exp OR 'sham procedure'/exp OR sham operation OR sham operations OR sham procedures OR sham therapies OR sham therapy OR sham treatment OR sham treatments OR 'placebo effect'/exp OR placebo reaction OR placebo response

'motor activity'/exp OR activity, motor OR 'exercise'/exp OR biometric exercise OR effort OR exercise capacity OR exercise performance OR exercise training OR exertion OR fitness training OR fitness workout OR physical conditioning, human OR physical effort OR physical exercise OR physical exertion OR physical work-out OR physical workout

'pain'/exp OR acute pain OR deep pain OR lightning pain OR nocturnal pain OR pain response OR pain syndrome OR treatment related pain OR 'nociception'/exp OR nociperception OR pain perception OR pain sensation OR pain sense OR pain sensitivity OR sensitivity, pain

'fatigue'/exp OR tiredness

'electrophysiology'/exp OR biopotential OR electrophysiological phenomena OR electrophysiological processes OR 'electrophysiological procedures'/exp electrophysiological recording OR electrophysiological technique OR electrophysiology technique

'systematic review (topic)'/exp OR systematic reviews OR systematic reviews as topic

===///============================

VHL

===///============================

(mh:(Efeito Placebo) OR (Resposta Placebo) OR (Resposta do Placebo) OR (Efecto Placebo) OR (Respuesta al Placebo) OR (Placebo Effect) OR (Effect, Placebo) OR (Effects,

Placebo) OR (Placebo Effects) OR (Placebo Response) OR (Response, Placebo) OR (HP3.073.433.101$) OR (N05.715.350.350.625$) OR (N06.850.490.734.875$) OR (SP5.001.012.038.059.075$)) OR (mh:(Efeito Nocebo) OR (Efecto Nocebo) OR (Nocebo Effect) OR (Effect, Nocebo) OR (Effects, Nocebo) OR (Nocebo) OR (Nocebo Effects) OR (N05.715.350.350.625.500$) OR (N06.850.490.734.875.500$)) OR (mh:(Placebos) OR (Placebo) OR (Placebos) OR (Placebos) OR (Sham Treatment) OR (D26.660$) OR (E02.785$) OR (HP3.073.433$) OR (HP7.007.130$))

(mh:(Atividade Motora) OR (Atividade Locomotora) OR (Actividad Motora) OR (Actividad Locomotora) OR (Motor Activity) OR (Activities, Motor) OR (Activity, Motor) OR (Motor Activities) OR (F01.145.632$) OR (G11.427.410.698$)) OR (mh:(Exercício Físico) OR (Atividade Física) OR (Atividade Física para Idoso) OR (Exercício) OR (Exercício Aeróbico) OR (Exercício Agudo) OR (Exercício Isométrico) OR (Treinamento Físico) OR (Ejercicio Físico) OR (Actividad Física) OR (Ejercicio) OR (Ejercicio Aeróbico) OR (Ejercicio Agudo) OR (Ejercicio Isométrico) OR (Entrenamiento Físico) OR (Exercise) OR (Activities, Physical) OR (Activity, Physical) OR (Acute Exercise) OR (Acute Exercises) OR (Aerobic Exercise) OR (Aerobic Exercises) OR (Exercise Training) OR (Exercise Trainings) OR (Exercise, Acute) OR (Exercise, Aerobic) OR (Exercise, Isometric) OR (Exercise, Physical) OR (Exercises) OR (Exercises, Acute) OR (Exercises, Aerobic) OR (Exercises, Isometric) OR (Exercises, Physical) OR (Isometric Exercise) OR (Isometric Exercises) OR (Physical Activities) OR (Physical Activity) OR (Physical Exercise) OR (Physical Exercises) OR (Training, Exercise) OR (Trainings, Exercise) OR (G11.427.410.698.277$) OR (I03.350$))

(mh:(Dor) OR (Sofrimento Físico) OR (Dolor) OR (Sufrimiento Físico) OR (Pain) OR (Ache) OR (Aches) OR (Burning Pain) OR (Burning Pains) OR (Crushing Pain) OR (Crushing Pains) OR (Migratory Pain) OR (Migratory Pains) OR (Pain, Burning) OR (Pain, Crushing) OR (Pain, Migratory) OR (Pain, Radiating) OR (Pain, Splitting) OR (Pains, Burning) OR (Pains, Crushing) OR (Pains, Migratory) OR (Pains, Radiating) OR (Pains, Splitting) OR (Physical Suffering) OR (Physical Sufferings) OR (Radiating Pain) OR (Radiating Pains) OR (Splitting Pain) OR (Splitting Pains) OR (Suffering, Physical) OR (Sufferings, Physical) OR (C23.888.592.612$) OR (F02.830.816.444$) OR (G11.561.790.444$)) OR (mh:(Percepção da Dor) OR (Percepción del Dolor) OR (Pain Perception) OR (Pain Perceptions) OR (Perception, Pain) OR (Perceptions, Pain) OR (F02.463.593.504$))

(mh:(Fadiga) OR (Lassitude) OR (Fatiga) OR (Lasitud) OR (Fatigue) OR (Lassitude) OR (C23.888.369$) OR (SP4.046.457.733$))

(mh:(Eletrofisiologia) OR (Electrofisiología) OR (Electrophysiology) OR (H01.158.344.528$) OR (H01.158.782.236$)) OR (mh:(Fenômenos Eletrofisiológicos) OR (Conceitos Eletrofisiológicos) OR (Processos Eletrofisiológicos) OR (Fenómenos Electrofisiológicos) OR (Conceptos Electrofisiológicos) OR (Procesos Electrofisiológicos) OR (Electrophysiological Phenomena) OR (Concept, Electrophysiologic) OR (Concept, Electrophysiological) OR (Concepts, Electrophysiologic) OR (Concepts, Electrophysiological) OR (Electrophysiologic Concept) OR (Electrophysiologic Concepts) OR (Electrophysiologic Phenomena) OR (Electrophysiological Concept) OR (Electrophysiological Concepts) OR (Electrophysiological Phenomenon) OR (Electrophysiological Process) OR (Electrophysiological Processes) OR (Phenomena, Electrophysiologic) OR (Phenomena, Electrophysiological) OR (Phenomenon, Electrophysiological) OR (Process, Electrophysiological) OR (Processes, Electrophysiological) OR (G07.265$))

((mh:(efeito placebo) OR (resposta placebo) OR (resposta do placebo) OR (efecto placebo) OR (respuesta al placebo) OR (placebo effect) OR (effect, placebo) OR (effects, placebo) OR (placebo effects) OR (placebo response) OR (response, placebo) OR (hp3.073.433.101*) OR (n05.715.350.350.625*) OR (n06.850.490.734.875*) OR (sp5.001.012.038.059.075*)) OR (mh:(efeito nocebo) OR (efecto nocebo) OR (nocebo effect) OR (effect, nocebo) OR (effects, nocebo) OR (nocebo) OR (nocebo effects) OR (n05.715.350.350.625.500*) OR (n06.850.490.734.875.500*)) OR (mh:(placebos) OR (placebo) OR (placebos) OR (placebos) OR (sham treatment) OR (d26.660*) OR (e02.785*) OR (hp3.073.433*) OR (hp7.007.130*))) AND ((mh:(atividade motora) OR (atividade locomotora) OR (actividad motora) OR (actividad locomotora) OR (motor activity) OR (activities, motor) OR (activity, motor) OR (motor activities) OR (f01.145.632*) OR (g11.427.410.698*)) OR (mh:(exercício físico) OR (atividade física) OR (atividade física para idoso) OR (exercício) OR (exercício aeróbico) OR (exercício agudo) OR (exercício isométrico) OR (treinamento físico) OR (ejercicio físico) OR (actividad física) OR (ejercicio) OR (ejercicio aeróbico) OR (ejercicio agudo) OR (ejercicio isométrico) OR (entrenamiento físico) OR (exercise) OR (activities, physical) OR (activity, physical) OR (acute exercise) OR (acute exercises) OR (aerobic exercise) OR (aerobic exercises) OR (exercise training) OR (exercise trainings) OR (exercise, acute) OR (exercise, aerobic) OR (exercise, isometric) OR (exercise, physical) OR (exercises) OR (exercises, acute) OR (exercises, aerobic) OR (exercises, isometric) OR (exercises, physical) OR (isometric exercise) OR (isometric exercises) OR (physical activities) OR (physical activity) OR (physical exercise) OR (physical exercises) OR (training, exercise) OR (trainings, exercise) OR (g11.427.410.698.277*) OR (i03.350*)) OR (mh:(dor) OR (sofrimento físico) OR (dolor) OR (sufrimiento físico) OR (pain) OR (ache) OR (aches) OR (burning pain) OR (burning pains) OR (crushing pain) OR (crushing pains) OR (migratory pain) OR (migratory pains) OR (pain, burning) OR (pain, crushing) OR (pain, migratory) OR (pain, radiating) OR (pain, splitting) OR (pains, burning) OR (pains, crushing) OR (pains, migratory) OR (pains, radiating) OR (pains, splitting) OR (physical

suffering) OR (physical sufferings) OR (radiating pain) OR (radiating pains) OR (splitting pain) OR (splitting pains) OR (suffering, physical) OR (sufferings, physical) OR (c23.888.592.612*) OR (f02.830.816.444*) OR (g11.561.790.444*)) OR (mh:(percepção da dor) OR (percepción del dolor) OR (pain perception) OR (pain perceptions) OR (perception, pain) OR (perceptions, pain) OR (f02.463.593.504*)) OR (mh:(fadiga) OR (lassitude) OR (fatiga) OR (lasitud) OR (fatigue) OR (lassitude) OR (c23.888.369*) OR (sp4.046.457.733*)) OR (mh:(eletrofisiologia) OR (electrofisiología) OR (electrophysiology) OR (h01.158.344.528*) OR (h01.158.782.236*)) OR (mh:(fenômenos eletrofisiológicos) OR (conceitos eletrofisiológicos) OR (processos eletrofisiológicos) OR (fenómenos electrofisiológicos) OR (conceptos electrofisiológicos) OR (procesos electrofisiológicos) OR (electrophysiological phenomena) OR (concept, electrophysiologic) OR (concept, electrophysiological) OR (concepts, electrophysiologic) OR (concepts, electrophysiological) OR (electrophysiologic concept) OR (electrophysiologic concepts) OR (electrophysiologic phenomena) OR (electrophysiological concept) OR (electrophysiological concepts) OR (electrophysiological phenomenon) OR (electrophysiological process) OR (electrophysiological processes) OR (phenomena, electrophysiologic) OR (phenomena, electrophysiological) OR (phenomenon, electrophysiological) OR (process, electrophysiological) OR (processes, electrophysiological) OR (g07.265*))) AND (db:("LILACS" OR "IBECS" OR "BRISA" OR "BINACIS" OR "CUMED" OR "BDENF" OR "LIPECS") AND type_of_study:("systematic_reviews"))

===///=========================

COCHRANE - 41

===///=========================

Date Run: **16/07/2021 15:33:45**

Comment:

ID	Search	Hits
**#1**	MeSH descriptor: [Placebo Effect] explode all trees	1609
**#2**	MeSH descriptor: [Nocebo Effect] explode all trees	40
**#3**	MeSH descriptor: [Placebos] explode all trees	24350
**#4**	MeSH descriptor: [Pain] explode all trees	51491
**#5**	MeSH descriptor: [Pain Perception] explode all trees	477
**#6**	MeSH descriptor: [Fatigue] explode all trees	3906

#7 MeSH descriptor: [Electrophysiological Phenomena] explode all trees 5570

#8 #1 OR #2 OR #3 AND (#4 OR #5 OR #6 OR #7) 4335

===///=============================

PROSPERO

===///=============================

#1

MeSH DESCRIPTOR PLACEBO EFFECT EXPLODE ALL TREES

#2

MeSH DESCRIPTOR Placebos EXPLODE ALL TREES

#3

MeSH DESCRIPTOR NOCEBO EFFECT EXPLODE ALL TREES

===///=============================

OATD

===///=============================

(("Placebo Effect" OR "Nocebo Effect" OR "Sham treatment") AND (Exercise OR Pain OR Pain Perception OR Fatigue OR Electrophysiology OR Electrophysiological Phenomena))
